# Book Reviews

**Published:** 1896-03

**Authors:** 


### *BOOK REVIEWS.*

The Pathology and Surgical Treatment of Tumors. By N. Senn, M.D., Ph.D., LL.D., Professor of Practice of Surgery and Clinical Surgery, Rush Medical College; Attending Surgeon of Presbyterian Hospital; Surgeon in Chief, St. Joseph's Hospital, Chicago. Illustrated by 515 Engravings, including full-page Colored Plate. Philadelphia: W. B. Saunders, 925 Walnut street.

Upon a careful reading of this work the impression is left that a vast deal of labor and research has been expended in its preparation. The great preponderance of data from foreign sources would indicate that comparatively little attention has been given here by surgeons to this kind of investigation, or that the author has overlooked their claims to his consideration. In a few instances it appears that home talent is not appreciated.

Taking a general view of the scope of this book, one is reminded of the announcement made by a speaker to his auditory, that he would treat of matters at the outset that they knew more about than he did; secondly, he would treat of matters that he was far better acquainted with than they were, and lastly he would discuss things that neither they nor he understood.

It is evident that surgeons who make any pretensions to familiarity with the gross features of tumors and have studied their macroscopic points with care, as many have done who undertake to teach surgery, will receive but little benefit from the general description of the different forms of tumors given in this work. Indeed, it may be justly claimed that, collectively, this class of readers know more about the characteristic outlines of tumors than the author has given in this volume.

On the other hand, the unmistakable evidence presented in this volume of the author's intimate acquaintance with the minute structure of tumors as revealed by the microscope and other means of investigation, places him in advance of others in knowledge of the organic elements of tumors. The failure to give the profession more light upon the ways and means of conducting these investigations, so as to arrive at correct conclusions, ought to be remedied in a future edition.

In regard to the origin of tumors not being in any way connected with the influence of bacteria, the position of the author is so widely at variance with his attitude as to the rôle of micro-organisms in nearly all inflammatory processes, that he is to be congratulated in excluding them from all participation in the etiological factor of tumors.

But just at this phase of development comes the great obstacle to a scientific explanation of the origin of neoplasms, and after all the elaborate investigations of original workers the profession is expected by the author to accept a theory, which is equally incomprehensible to writer and reader.

After considerable labor in connection with this whole matter, some years ago by the writer, it was found that no more expressive definition could be given than this: "A tumor is an abnormal development of tissue independent of the ordinary results of inflammation." Whether the cell-division theory or that of Conheim may be accepted, there is no practical advance made in comprehending the development of tumors. Hence, it is a fair deduction that the author finds a problem as to etiology which is unintelligible to all concerned. His definition is, however, stated very emphatically, that "a tumor is a localized increase of tissue, the product of tissue-proliferation of embryonic cells of congenital or post-natal origin, produced independently of microbic causes."

The division of embryonic tissue into epiblast, hypoblast, and mesoblast, is made to aid the student in comprehending the location and structure of tumors. The author has classified under the general heading of epiblastic and hypoblastic tumors: papilloma, adenoma, cystoma, and carcinoma; under that of mesoblastic tumors: fibroma, lipoma, myxoma, chondroma, osteoma, angioma, lymphangioma, lymphoma, myoma, neuroma, and sarcoma; under combinations of the above comes terratoma, and he closes with retention cysts.

The elaborate and exhaustive consideration of each of these topics, with numerous illustrations, sustains the reputation for exactness and thoroughness which the author has established by his previous publications.

TWENTIETH CENTURY PRACTICE. An International Encyclopedia of Modern Medical Science. By leading authorities of Europe and America. Edited by Thomas L. Stedman, M.D., New York City. In twenty volumes. Volume VI. Diseases of the Respiratory Organs. William Wood & Company, 45–47 East Tenth street, New York.

The sixth volume of this excellent work appears ahead of the fifth on account of some unavoidable delay in receipt of manuscript for the latter. The volume before us is devoted to diseases of the respiratory tract, and these are treated of in a very complete and satisfactory manner. The opening article is naturally one upon the "Diseases of the Nose." This is from the pen of Dr. Prosser James, of London, the possessor of a graceful literary style, who has given us one of the most readable articles in the book. Following this is an article on the "Diseases of the Accessory Sinuses of the Nose," by Dr. Jonathan Wright, of Brooklyn. Two articles, one on "Diseases of the Naso-Pharynx and Pharynx," and the other on the "Diseases of the Tonsils," are by Dr. E. J. Moure, of Bordeaux, who is recognized as the leading French authority on affections of the upper air passages. Dr. A. H. Buck, of New York, treats of "Diseases of the Ears," in his usual clear and easy style. Proceeding downward, we come to the "Larynx," the diseases of which are handled in a very satisfactory manner by Dr. F.H. Bosworth, of New York. All of the affections of the upper air passages just mentioned are treated from the point of view of the general practitioner rather than from that of the specialist. Sir Thomas Grainger Stewart and Dr. George A. Gibson, of Edinburgh, have written in collaboration upon the "Diseases of the Trachea and Bronchial Tubes," the result of their combined labors being an excellent treatise on the pathology and treatment of these harassing complaints. The author of the concluding article is Dr. Winslow Anderson, of San Francisco. His subject is the "Diseases of the Lungs," except tuberculosis and croupous pneumonia, two affections which will be considered in a later volume along with the other infectious diseases.

As regards both the interest of the subjects here treated and the authority of the writers, this volume will compare favorably with any of its predecessors. "Diseases of the Skin" will form the contents of the next volume.

THE AMERICAN YEAR-BOOK OF MEDICINE AND SURGERY. Prepared and arranged by eminent American specialists and teachers, under the editorial charge of George M. Gould, M.D., Philadelphia. Illustrated with numerous wood-cuts, half-tones, and colored plates. Price, cloth, $6.50. Published by W. B. Saunders, 925 Walnut street, Philadelphia.

This book has been prepared by the contributors and publisher from the desire to furnish the general physician with a carefully-made epitomization of the most important current journalistic literature for the year ending June 30, 1895. The object has been to include only what is new, new ideas of medical treatment and new methods of surgical practice. Among the contributors are such well-known names as William Pepper, W. W. Keen, Louis Starr, V. P. Gibney, C. H. Burnett, Archibald Church, W. A. Hardaway, and others, and the subjects discussed and brought down to the date named are, General Medicine, Surgery, Obstetrics, Gynecology, Pediatrics, Nervous Diseases, Dermatology, Orthopedics, Ophthalmology, Otology, Rhinology and Laryngology, Pathology and Bacteriology, Materia Medica and Therapeutics, Anatomy and Physiology, and Hygiene and Chemistry. All the important literature bearing on these subjects has been carefully gone over and digested, and due mention has been made of all distinct progress in our medical and surgical knowledge and technique. The contributors have made an eminent success of this work, and we think it will be read with interest and profit by all subscribers. The fine editorial hand of Dr. Gould is well apparent throughout the book.

---

COLOR VISION AND COLOR BLINDNESS. A Practical Manual for Railroad Surgeons. By J. Ellis Jennings, M.D., University of Pennsylvania. The F. A. Davis Company, Publishers, Philadelphia, Pa.

This little book has just been issued from the above publishing house, and Dr. Jennings has put in a very clear and practical form just those points about color vision and blindness which are most needed by the student of ophthalmology. One of the incentives for writing this book is given by Dr. Jennings in his preface: "The methods now employed have proven practical and efficient, and there is every reason to believe that all the railroads in this country would take measures to weed out the color-blind from the service if the frequency and dangers of this affection were brought to their notice. It is with the hope of stimulating further effort in this direction that this manual has been written."

The rules regulating the employment of men whose vision and color perception have been thoroughly tested are much more strictly carried out by the management of the large Northern railroads than is found here in the South. For instance, on the magnificently regulated Pennsylvania railroad, no man can be employed whose vision for color perception is defective. It is certainly not due to the fact that our Northern brethren value human lives more than we of the South that such a provision is made for the safety of railroad travel, but either through ignorance or a miserly desire to reduce every expense, there is scarcely any regulation among the railroads in this section for any examination whatever of their employees in regard to vision or color perception. In some States there are laws regulating railroads in regard to this point, and we sincerely trust that such a movement may prove more universal. For oculists, consultants to railroads, where such regulations are demanded, the little book of Dr. Jennings will prove a safe and valuable guide. D. R.

---

THE HISTORY OF ANESTHESIA. By William R. Hayden, M.D., Bedford Springs, Massachusetts. Price 25 cents. [Reprinted from the *International Journal of Surgery*.]

The author begins this brochure by saying that in presenting the important facts bearing upon the discovery of anesthesia, he will put forth an "honest effort to be impartial and just, as the truth demands." In spite of such an avowed purpose he has given a distorted and one-sided story, in which W. T. G. Morton appears as the particular hero, and the claims of Long and Wells and Jackson to part of the credit of this discovery are repudiated as unworthy of consideration. Dr. Hayden's mind has never been easy since it has been proven beyond question that Dr. Long, of Georgia, was the first to employ surgical anesthesia by ether in 1842. Within the last two years he has written seven or eight journal articles in the interest of Dr. Morton, and we have reason to believe that the end is not yet. *Quousque tandem, Catilina, abutere patientia nostra.*

But not all the prejudice and false logic of Dr. Hayden, nor the interpretation which he is pleased to place upon the work and motives of Dr. Long can eradicate the single fact that the latter was acquainted with the anesthetic properties of sulphuric ether and had several times operated thereby four years before Dr. Morton ever thought of it. All of the claimants to the credit of the discovery of anesthesia have had their cases fully presented, and upon the data thus presented the final judgment will be made up, awarding each claimant his proper due.

---

### DON'TS FOR CONSUMPTIVES, OR THE SCIENTIFIC MANAGEMENT OF PULMONARY TUBERCULOSIS.

This is the title of a book which, under the authorship of Dr. Charles Wilson Ingraham, will soon be issued by the Medical Reporter Publishing Co. of Rochester, N. Y.

The complete work of thirty-five chapters is devoted exclusively to the general management of Pulmonary Invalids, no reference whatever being made to drug treatments.

The object of the author is to supply the physician with a practical work, and at the same time, by eliminating technical terms, reduce the text within the easy comprehension of the intelligent patient. The author claims that "a good understanding of his condition is the best remedy for the consumptive." With this book in the hands of his patient, the physician will be relieved of a multitude of details which attach to the successful management of such cases. Special attention has been given those chapters pertaining to the destruction of tubercular infection.

The book will be printed on 72-pound antique book paper, bound in cloth (imitation morocco), with title in gold leaf. Price, $1.75.

